# Supplementary material for: NMR-Based Metabolic Profiling of the Effects of α-Ketoglutarate Supplementation on Energy-Deficient C2C12 Myotubes
Source: Molecules. 2023 Apr 30;28(9):3840. doi: 10.3390/molecules28093840 (PMC10179873; doi:10.3390/molecules28093840)
Supplement: Supplementary file 1 [file molecules-28-03840-s001.zip › molecules-2314530-supplementary.pdf]

Supplementary Materials

Figure S1.

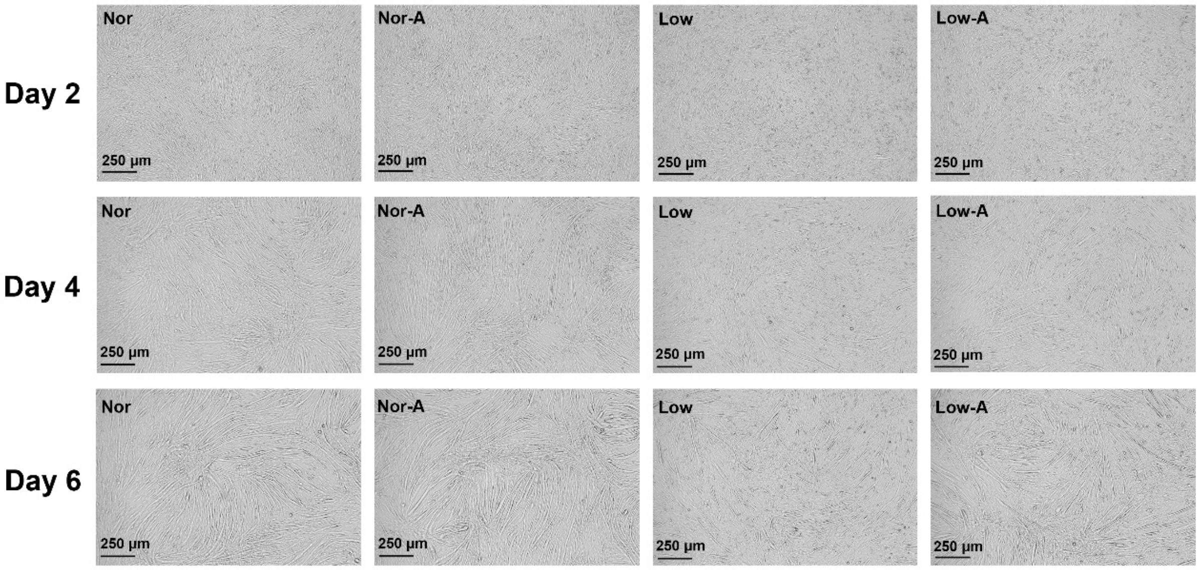

Figure S1. Morphologies of the Nor, Nor-A, Low and Low-A groups of C2C12 myotubes.

Figure S2.

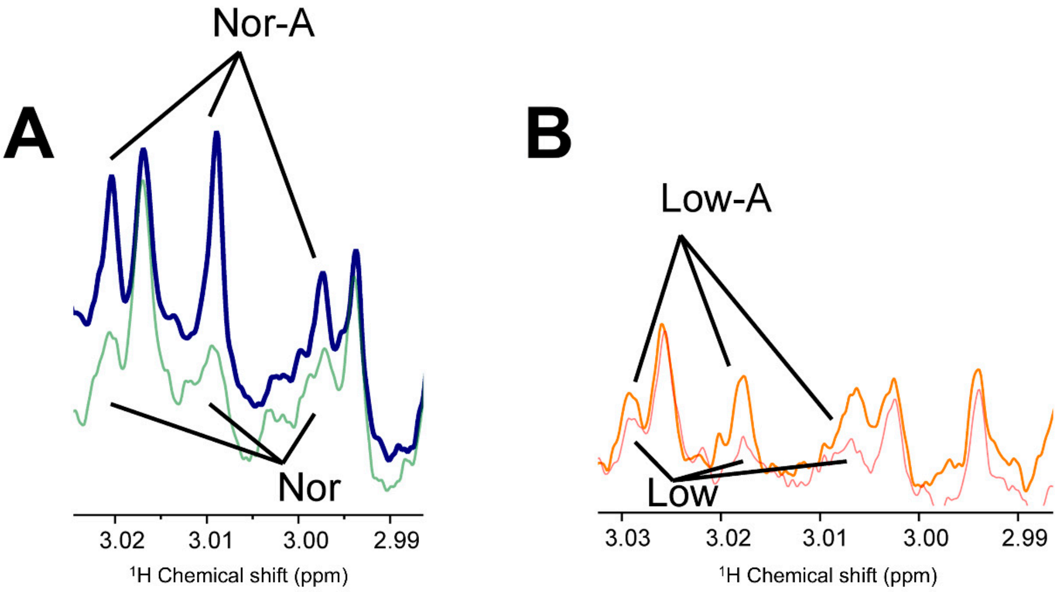

Figure S2. Local amplified regions of AKG peaks in 1D ^{1}H-NMR spectrum.

Figure S3.

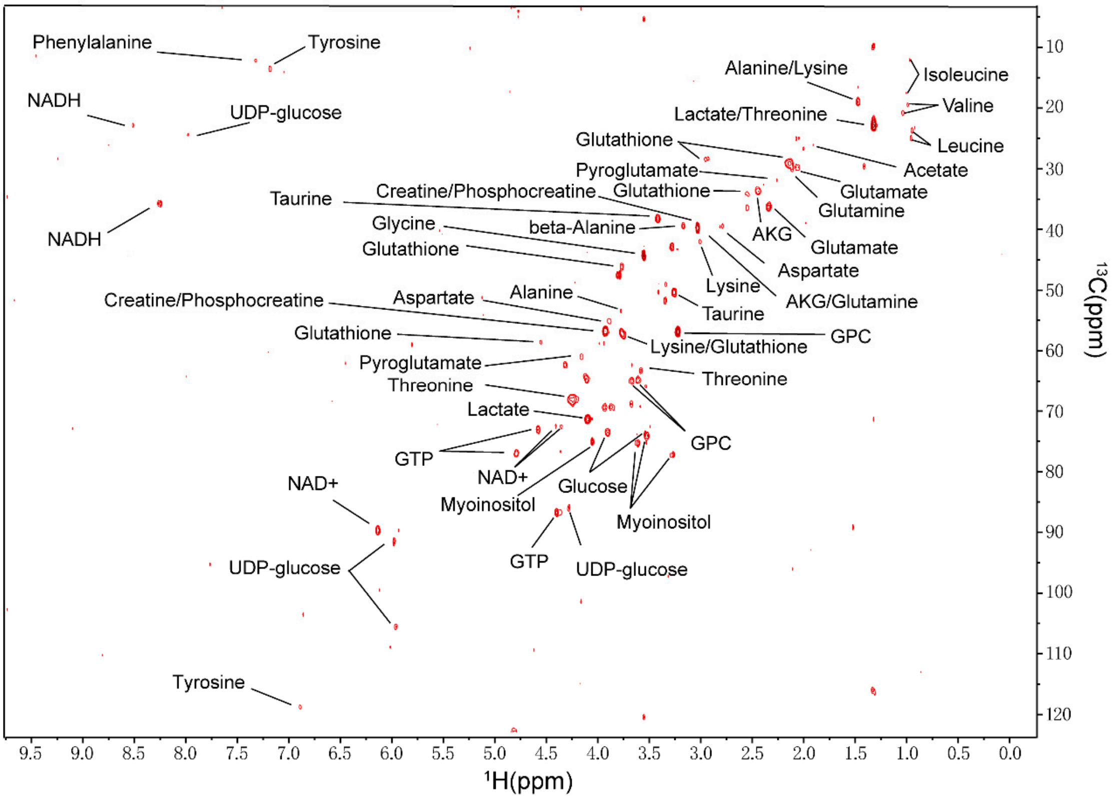

Figure S3. Representative 2D ^{1}H-^{13}C HSQC spectrum of aqueous extracts from C2C12 myotubes recorded on a 600 MHz NMR spectrometer.

Figure S4.

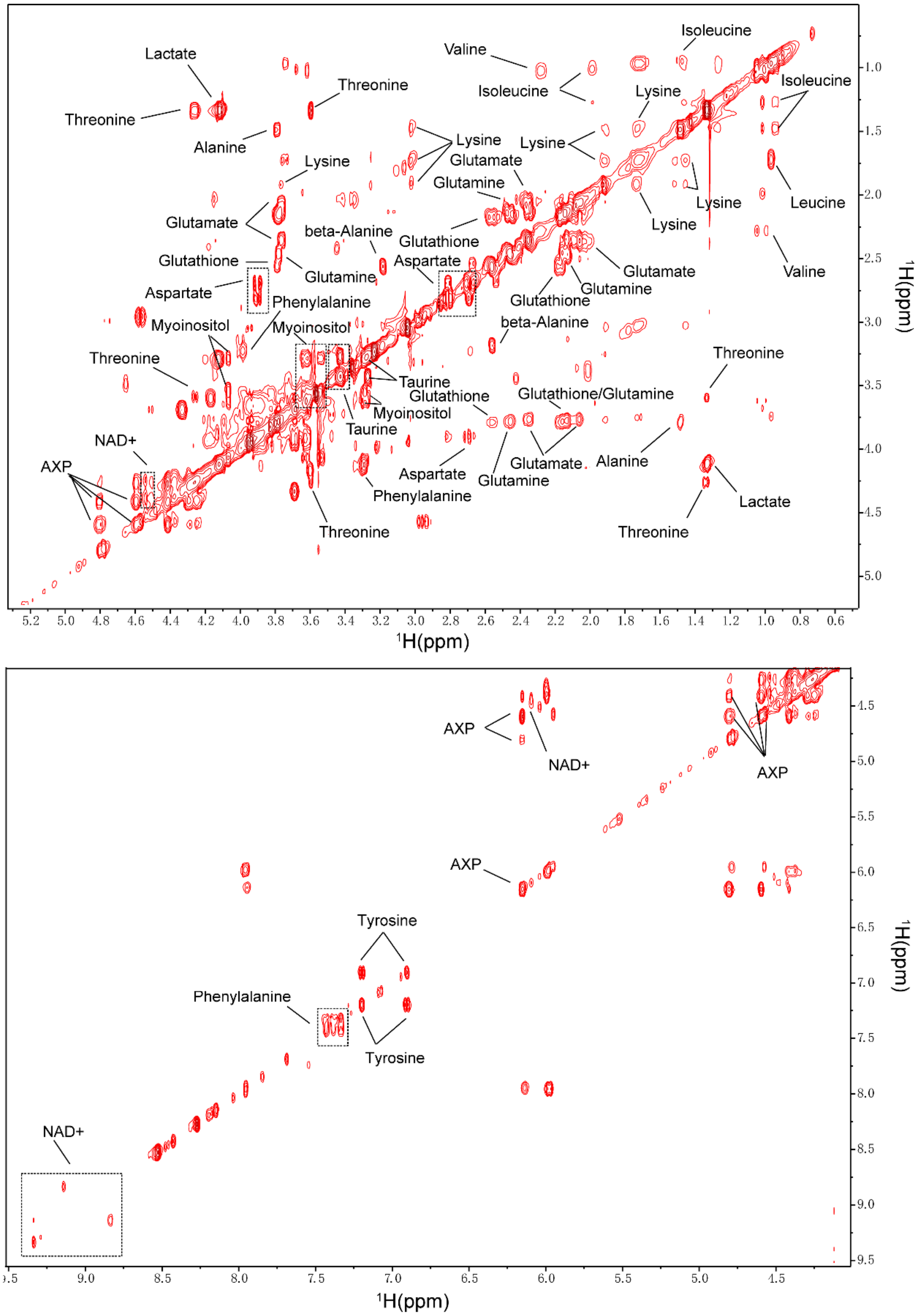

Figure S4. Representative 2D ^{1}H-^{1}H TOCSY spectrum of aqueous extracts from C2C12 myotubes recorded on a 600 MHz NMR spectrometer.

Figure S5.

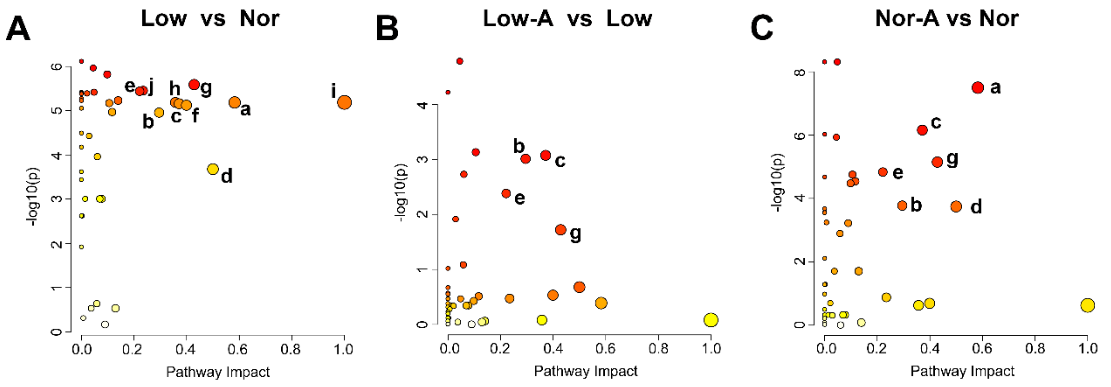

Figure S5. Significantly altered metabolic pathways of Low *vs.* Nor, Low-A *vs.* Low, Nor-A *vs.* Nor. The letters in the panels represent significantly altered metabolic pathways: a. Alanine, aspartate and glutamate metabolism; b. Glycine, serine and threonine metabolism; c. Glutathione metabolism; d. D-Glutamine and D-glutamate metabolism; e. Histidine metabolism; f. beta-Alanine metabolism; g. Taurine and hypotaurine metabolism; h. Phenylalanine metabolism; i. Phenylalanine, tyrosine and tryptophan biosynthesis; j. Nicotinate and nicotinamide metabolism.

Table S1.

Table S1. Resonance assignments of aqueous extracts from C2C12 myotubes.

No.	Metabolite	δ ^{1}H (ppm) and multiplicity	Moieties
1	Leucine	0.96 (d), 0.97 (d), 1.69 (m), 1.70 (m), 1.73 (m), 3.73 (m)	α-CH_3, α-CH_3, γ-CH, β-CH_2, α-CH
2	Isoleucine	0.94 (t), 1.01 (d), 1.21 (m), 1.42 (m), 2.00 (m), 3.67 (d)	δ-CH_3, γ-CH_3, half γ-CH_2, half γ-CH_2, β-CH, α-CH
3	Valine	0.99 (d), 1.05 (d), 2.26 (m), 3.60 (d)	γ-CH_3, γ-CH_3, β-CH, α-CH
4	Ethanol	1.17 (t), 3.65 (q)	δ-CH_3, CH_2
5	Threonine	1.33 (d), 3.58 (d), 4.24 (m)	γ-CH_2, β-CH
6	Lactate	1.33 (d), 4.11 (q)	β-CH_3, α-CH
7	Alanine	1.47 (d), 3.78 (q)	β-CH_3, α-CH
8	Acetate	1.91 (s)	CH_3
9	Pyroglutamate	2.05 (m), 2.39 (d), 2.51 (m), 4.18 (dd)	β-CH, γ-CH_2, β-CH, α-CH
10	Glutamate	2.08 (m), 2.12 (m), 2.34 (m), 2.37 (m), 3.75 (m)	Half β-CH_2, half β-CH_2, half γ-CH_2, half γ-CH_2, α-CH

11	Lysine	1.43 (m), 1.49 (m),1.70 (m), 1.91 (m), 3.02(t), 3.75 (t)	half γ-CH_2, half γ-CH_2, δ-CH_2, β-CH_2, ε-CH_2, α-CH
12	Glutamine	2.13 (m), 2.45 (m), 3.77 (t)	γ-CH_2, β-CH_2, α-CH
13	Glutathione	2.15 (m), 2.55 (m), 2.96 (m), 3.77 (m), 4.56 (m)	β-CH_2, γ-CH_2, CH_2-SH, α-CH &CH_2–NH, CH–NH
14	α-Ketoglutarate (AKG)	2.45 (t), 3.01 (t)	CH_2, CH_2
15	beta-Alanine	2.54 (t), 3.17 (t)	CH_2, CH_2
16	Aspartate	2.68 (dd), 2.81 (dd), 3.90 (dd)	β-CH_2, α-CH
17	Creatine	3.04 (s), 3.93 (s)	N-CH_3, CH_2
18	Phosphocreatine	3.05 (s), 3.94 (s)	N-CH_3, CH_2
19	Tyrosine	3.05 (dd), 3.19 (dd), 6.92 (d), 7.19 (d)	half β-CH_2, half β-CH_2, β-CH, α-CH
20	Phenylalanine	3.12 (dd), 3.30 (dd), 3.99 (dd), 7.33 (d), 7.37 (t), 7.43 (t)	α-CH, half β-CH_2, half β-CH_2, α-CH, β-CH, γ-CH
21	sn-Glycero-3-phosp-hocholine(GPC）	3.23 (s), 3.60 (dd), 3.68 (dd), 3.87 (m), 3.94 (m), 4.33 (m)	N–$(CH_3)_3$, half $^1CH_2$, $^2CH_2$, half $^2CH_2$, half $^3CH_2$, half $^3CH_2$, $^1CH_2$
22	Taurine	3.24 (t), 3.41 (t)	$^1CH_2$, $^2CH_2$
23	Myo-inositol	3.2(t), 3.53(dd), 3.63(t), 4.07(t)	^{2}CH, ^{4,6}CH, ^{1,3}CH, ^{5}CH
24	Glucose	β (3.24 (dd), 3.48 (t), 3.90 (dd)), α (3.54 (dd), 3.71 (t), 3.72 (dd), 3.83 (m))	β (H_2, H_3, H_5), α (H_2, H_3, H_6)
25	Glycine	3.57 (s)	α-CH_2
26	UDP-glucose	5.62 (dd), 6.0 (m）	CH, ^{2}CH
27	GTP	5.92 (d), 8.1 (s）	CH, CH
28	NAD+	6.03 (d), 6.08 (s), 8.16 (s), 8.20 (m), 8.41 (s), 8.82 (d), 9.13 (d), 9.32 (s)	NH_2, NH_2(CO), δ-CH, β-CH, ^{2}CH, γ-CH, α-CH
29	NADH	8.23 (s), 8.47 (s)	γ-CH, α-CH
30	AXP	6.14 (d), 8.27 (s), 8.58 (s)	NH_2, δ-CH, ^{2}CH
31	Histidine	7.06 (s), 7.85 (s)	^{5}CH, ^{2}CH
32	3-Methylhistidine	7.03 (s), 7.93 (s)	^{5}CH, ^{2}CH
33	Formate	8.46 (s)	CH

[a] Multiplicity: s, singlet; d, doublet; t, triplet; q, quartet; m, multiplet; dd, doublet of doublets.

Table S2.

Table S2. Comparison of relative metabolite levels between the Nor, Nor-A, Low and Low-A groups of C2C12 myotubes on the basis of relative NMR integrals.

**Metabolites**	**Mean ± Standard error**				One-way ANOVA		pairwise comparisons		
	**Nor**	**Nor-A**	**Low**	**Low-A**	**F**	***p***	Nor-A *vs.* Nor	Low *vs.* Nor	Low-A *vs.* Low
Leucine	0.553 ± 0.005	0.554 ± 0.008	1.056 ± 0.011	1.072 ± 0.037	291.647	< 0.0001	ns	****	ns
Isoleucine	0.354 ± 0.004	0.354 ± 0.006	0.648 ± 0.008	0.643 ± 0.012	490.611	< 0.0001	ns	****	ns
Valine	0.356 ± 0.004	0.358 ± 0.006	0.689 ± 0.007	0.678 ± 0.009	859.224	< 0.0001	ns	****	ns
Ethanol	0.164 ± 0.016	0.151 ± 0.013	0.100 ± 0.013	0.102 ± 0.017	4.972	0.007	ns	**	ns
Alanine	1.495 ± 0.023	1.265 ± 0.019	0.479 ± 0.014	0.465 ± 0.021	726.993	< 0.0001	ns	****	ns
Acetate	0.044 ± 0.002	0.044 ± 0.001	0.055 ± 0.002	0.062 ± 0.003	18.570	< 0.0001	ns	***	ns
Glutathione	0.212 ± 0.001	0.184 ± 0.005	0.207 ± 0.003	0.202 ± 0.004	11.749	< 0.0001	****	ns	ns
Glutamate	1.412 ± 0.024	1.319 ± 0.016	3.683 ± 0.058	3.646 ± 0.072	905.085	< 0.0001	**	****	ns
AKG	0.132 ± 0.006	0.166 ± 0.006	0.154 ± 0.011	0.200 ± 0.023	22.516	< 0.0001	**	ns	ns
Pyroglutamate	0.400 ± 0.005	0.405 ± 0.004	0.450 ± 0.015	0.504 ± 0.015	5.234	0.005	ns	**	*
Glutamine	1.377 ± 0.014	1.351 ± 0.012	1.338 ± 0.035	1.275 ± 0.040	2.544	0.076	ns	ns	ns
Asparate	0.218 ± 0.007	0.237 ± 0.005	2.041 ± 0.047	2.002 ± 0.050	1096.978	< 0.0001	*	****	ns
Lysine	0.117 ± 0.007	0.122 ± 0.007	0.178 ± 0.014	0.178 ± 0.025	5.954	0.003	ns	**	ns
Creatine	2.767 ± 0.027	2.880 ± 0.034	3.289 ± 0.067	3.393 ± 0.058	41.297	< 0.0001	*	****	ns
Phosphocreatine	1.717 ± 0.020	1.808 ± 0.017	3.144 ± 0.047	3.224 ± 0.032	736.275	< 0.0001	**	****	ns
Beta-Alanine	0.526 ± 0.009	0.536 ± 0.009	0.788 ± 0.023	0.836 ± 0.035	66.141	< 0.0001	ns	****	ns
GPC	3.080 ± 0.031	3.632 ± 0.038	6.525 ± 0.043	6.381 ± 0.149	641.068	< 0.0001	****	****	ns
Taurine	1.668 ± 0.013	1.812 ± 0.018	3.049 ± 0.024	2.813 ± 0.091	282.066	< 0.0001	****	****	*
MyoInositol	0.482 ± 0.016	0.439 ± 0.005	0.536 ± 0.069	0.545 ± 0.015	1.999	0.136	*	ns	ns

Glucose	0.055 ± 0.008	0.080 ± 0.011	0.003 ± 0.000	0.003 ± 0.000	26.694	< 0.0001	ns	****	ns
Glycine	7.766 ± 0.062	8.414 ± 0.087	4.125 ± 0.033	4.802 ± 0.069	1005.889	< 0.0001	****	****	****
Lactate	3.735 ± 0.020	3.740 ± 0.020	0.519 ± 0.015	0.430 ± 0.012	########	< 0.0001	ns	****	***
Threonine	1.527 ± 0.013	1.263 ± 0.061	1.215 ± 0.062	1.074 ± 0.058	13.463	< 0.0001	***	****	ns
GTP	0.139 ± 0.002	0.152 ± 0.002	0.138 ± 0.006	0.138 ± 0.004	3.482	0.028	***	ns	ns
UDP-Glucose	0.020 ± 0.001	0.021 ± 0.002	0.006 ± 0.001	0.006 ± 0.001	50.512	< 0.0001	ns	****	ns
Tyrosine	0.146 ± 0.002	0.147 ± 0.002	0.263 ± 0.003	0.262 ± 0.004	565.996	< 0.0001	ns	****	ns
Phenylalanine	0.367 ± 0.005	0.354 ± 0.005	0.707 ± 0.019	0.720 ± 0.013	311.856	< 0.0001	ns	****	ns
Histidine	0.023 ± 0.001	0.023 ± 0.001	0.039 ± 0.001	0.042 ± 0.001	22.914	< 0.0001	ns	****	ns
π-Methylhistidine	0.027 ± 0.001	0.036 ± 0.001	0.026 ± 0.002	0.039 ± 0.002	105.721	< 0.0001	****	ns	***
NAD+	0.052 ± 0.001	0.052 ± 0.001	0.081 ± 0.001	0.078 ± 0.002	184.371	< 0.0001	ns	****	ns
NADH	0.016 ± 0.001	0.019 ± 0.001	0.003 ± 0.001	0.002 ± 0.001	129.831	< 0.0001	ns	****	ns
Formate	0.016 ± 0.001	0.018 ± 0.001	0.040 ± 0.002	0.042 ± 0.001	99.695	< 0.0001	*	****	ns
AXP	0.670 ± 0.007	0.720 ± 0.008	0.826 ± 0.007	0.808 ± 0.011	82.707	< 0.0001	***	****	ns

[a] The relative levels of the metabolites were quantified on the basis of the NMR integrals normalized to the total integrals.

[b] Statistical significances (p values) were calculated from One-way ANOVA: * $p < 0.05$, ** $p < 0.01$, *** $p < 0.001$, **** $p < 0.0001$ Red/blue colors denote increased/ decreased metabolites, respectively.

Table S3. Relevant information of significantly altered metabolic pathways and matched metabolites.

No.	Metabolic Pathway	PIV	Match Status	Matched metabolites	*p* value		
					Low vs. Nor	Low-A vs. Low	Nor-A vs. Nor
P1	Alanine, aspartate and glutamate metabolism	0.583	5/28	Alanine, Aspartate, Glutamate, AKG, Glutamine,	6.532×10^{-6}	0.408	3.207×10^{-8}
P2	Glycine, serine and threonine metabolism	0.295	3/34	Threonine, Glycine, Creatine,	1.108×10^{-5}	9.802×10^{-4}	1.683×10^{-4}
P3	Glutathione metabolism	0.371	4/28	Glycine, Glutathione, Glutamate, 5-Oxoproline	6.968×10^{-6}	8.471×10^{-4}	6.974×10^{-7}
P4	D-Glutamine and D-glutamate metabolism	0.500	3/6	Glutamine, Glutamate, AKG	2.100×10^{-4}	0.210	1.794×10^{-4}
P5	Histidine metabolism	0.221	4/16	Histidine, Glutamate, N(pi)-Methyl-L-histidine, Aspartate	3.642×10^{-6}	0.004	1.469×10^{-5}
P6	beta-Alanine metabolism	0.399	3/21	Aspartate, beta-Alanine, Histidine	7.538×10^{-6}	0.294	0.204
P7	Taurine and hypotaurine metabolism	0.429	1/8	Taurine	2.579×10^{-6}	0.019	7.108×10^{-6}
P8	Phenylalanine metabolism	0.357	2/12	Phenylalanine, Tyrosine	6.506×10^{-6}	0.830	0.236
P9	Phenylalanine, tyrosine and tryptophan Biosynthesis	1.000	2/4	Phenylalanine, Tyrosine	6.506×10^{-6}	0.830	0.236
P10	Nicotinate and nicotinamide metabolism	0.235	2/15	Aspartate, NAD+	3.526×10^{-6}	0.337	0.132

[a] Metabolic pathway analysis was performed on the MetaboAnalyst 5.0 webserver, using a combination of metabolite sets enrichment analysis with a criterion of statistical significance $p < 0.05$ and pathway topological analysis with a criterion of pathway impact value (PIV) > 0.2.
